# Imbalance of Bile Acids Metabolism Mediated by Gut Microbiota Contributed to Metabolic Disorders in Diabetic Model Mice

**DOI:** 10.3390/biology14030291

**Published:** 2025-03-13

**Authors:** Hongwang Dong, Xinguo Liu, Ge Song, Wenting Peng, Xihan Sun, Wei Fang, Wentao Qi

**Affiliations:** 1 Key Laboratory of Grain and Oil Biotechnology, Academy of National Food and Strategic Reserves Administration, Beijing 100037, China; dhw405@126.com (H.D.); lxgg00@163.com (X.L.); sg@ags.ac.cn (G.S.); pwt@ags.ac.cn (W.P.); sxh@ags.ac.cn (X.S.); 2 College of Food Science and Engineering, Central South University of Forestry and Technology, National Engineering Research Center for Rice and Byproduct Deep Processing, Changsha 410004, China

**Keywords:** type 2 diabetes, db/db mice, bile acids, intestinal flora

## Abstract

Type 2 diabetes (T2D) is a chronic disease prevalent in the world, accompanied by a variety of diseases, endangering human health and safety. Bile acids (BAs) play an important role in the regulation of host glucose and lipid metabolism homeostasis, and are strictly regulated by gut microbiota. However, the relationship between key BAs, BAs transporters and signaling, as well as gut microbiota, and host metabolism in T2D remains elusive. In this study, 9-week-old db/db mice were used as diabetes model (db/db group, *n* = 10), and their wild-type (wt) littermates of same age were used as the healthy control (CON group, *n* = 10). After 8 weeks of feeding, the BA profiles and microbial composition in the colon, and gene expression level of BA regulatory factors were analyzed in the db/db and CON groups to explore the underlying mechanisms of T2D. Compared with healthy mice, the body weight, blood glucose and lipid levels of db/db mice were significantly increased. The concentrations of total BAs, primary BAs, conjugated BAs and non-12α–hydroxylated BAs (non-12–OH BAs) were significantly decreased, while Deoxycholic acid (DCA) in secondary BAs was increased in db/db group. Compared with wt mice, the synthesis of BAs in the liver was transformed from the alternative pathway to the classical pathway, and hepatic BAs transporters (NTCP, BSEP, MRP2, OATP–1 and OSTβ) and receptors (FXR and TGR5) were significantly down-regulated in the db/db mice. In the colon, the mRNA level of FXR was up-regulated, while TGR5 was down-regulated. The diabetic (db/db) mice presented a changed gut microbiota composition, including an increased abundance of secondary BAs-producing bacteria, *Escherichia–Shigella*, and a decreased the abundance of *Akkermansia*, which are involved in the synthesis of non-12–OH BAs. We further found that the reduced BA types in db/db mice were negatively correlated with metabolic-disorder-related indicators, while an increased DCA level had the opposite correlation. Our results shed light into how the imbalance of BAs’ metabolism mediated by intestinal flora may be potential mechanisms of T2D.

## Simple Summary

Type 2 diabetes (T2D) is a chronic disease prevalent in the world, endangering human health and safety. Concurrently, there has been a growing concern among individuals regarding the association of the pathogenesis of T2D with bile acids (BAs) and intestinal flora. However, the relationship between key BAs, BAs transporters and signaling, as well as gut microbiota, and host metabolism in T2D remains elusive. In this study, we found that all BAs of diabetes model mice showed a decreasing trend except for DCA compared to healthy mice, resulting in the disruption of BAs’ transporter expression and signaling. Additionally, it altered the diversity and composition of gut bacteria and promoted the generation of secondary BAs and non-12–hydroxylated BAs. These findings indicate that the disruption of BAs’ metabolism mediated by gut microbiota may be a potential mechanism of T2D. Our article aims to facilitate the understanding of T2D pathogenesis.

## 1. Introduction

Type 2 diabetes (T2D) has become a serious chronic disease threatening human health worldwide, which is an exceedingly formidable challenge to public health. The prevalence of T2D affects more than 400 million people worldwide, and the global incidence of T2D is expected to increase to 700 million by 2045 [1,2]. The characteristic symptoms of T2D entail elevated fasting blood glucose level and increased plasma total cholesterol, triglycerides, and LDL cholesterol [3]. Several studies have concluded that the pathogenic mechanism of T2D is more complex, involving environmental, genetic, dietary and lifestyle factors, leading to defective pancreatic islet B-cell function and insulin resistance associated with decreased sensitivity to glucose, contributing to the rise in blood glucose [4].

More recently, the intricate relationship between bile acids (BAs) and the pathogenesis of T2D has gained significant attention [5]. BAs are the end products of cholesterol metabolism by the liver, and their synthesis, transport, and excretion homeostat play a crucial role in regulating glucose and lipid metabolism balance. BAs are synthesized in the liver through two routes, one mediated by Cholesterol 7α-hydroxylase (CYP7A1) and controlled by Sterol 12α-hydroxylase (CYP8B1), leading to the classical pathway producing the 12α-hydroxy primary BA, Cholic acid (CA). The other is an alternative pathway, catalyzed by Sterol 27-hydroxylase (CYP27A1), further hydroxylated by Oxysterol 7α-hydroxylase (CYP7B1), primarily producing non-12α-hydroxylated primary BA, Chenodeoxycholic acid (CDCA), which is converted to MCA in mice [6]. The conjugation of primary BAs with taurine or glycine is facilitated by the action of Bile acyl-CoA synthetase (BACS) and Bile acid-CoA: amino acid N-acyltransferase (BAAT), resulting in the formation of conjugated BAs, before their secretion into bile via the Bile salt export pump (BSEP) and Multidrug resistance-associated protein 2 (MRP2) [7,8,9]. In addition, BAs transport proteins that regulate the enterohepatic circulation of BAs and maintain their normal physiological functions, such as, multidrug resistance-associated protein 3 (MRP3), organic solute transporter α (OSTα) and organic solute transporter β (OSTβ), which excrete excess hepatic BAs into the systemic circulation, and sodium taurocholate cotransporting pol-ypeptide (NTCP) and organic anion transporting polypeptide-1 (OATP–1), which are responsible for the recirculation of ileal BAs to the liver to maintain BAs’ pool homeostasis [8,10]. Meanwhile, BAs can also act as signaling molecules, targeting Farnesoid X receptor (FXR) and Takeda G protein-coupled receptor 5 (TGR5) on the cell nucleus or cell membrane and regulating glucose, lipid homeostasis, and energy expenditure [11].

The gut microbiota participates in BAs’ metabolic homeostasis. The primary BAs synthesized by the liver are converted to secondary BAs in the gut and undergo four key modifications by the intestinal microbiota: deconjugation, 7α-dehydroxylation, oxidation, and epimerization [12]. Moreover, the alternative pathway of BA synthesis might be regulated by intestinal flora. Increased 12α–Hydroxy bile acids (12–OH BAs) levels were found to be associated with *Akkmermansia*, *Bacteroides* and *Clostridium scindens* [13]. Therefore, the interaction between BAs and intestinal flora is of great significance for the occurrence and development of human health [14].

Studies have reported that in diabetic individuals and model animals, the serum and fecal BAs’ composition and intestinal flora were abnormal [15,16], suggesting that BAs might be a main target affecting glucose metabolism. However, the data on the relationship between intestinal BAs profile, BAs signaling and intestinal flora in T2D are limited, and more studies still need to be conducted. db/db mice are regarded as the most proximate animal model to T2D [17]. Therefore, intestinal BAs’ profiles and liver BAs’ synthesis, transport and BAs’ receptor regulation, as well as intestinal flora composition, were investigated using db/db mice. This study aims to provide information on the bile acids–intestinal flora axis in db/db mice, which may facilitate the understanding of T2D pathogenesis.

## 2. Materials and Methods

### 2.1. Chemicals and Reagents

Cholesterol (TC), triglyceride (TG), high-density lipoprotein cholesterol (HDL–C), low-density lipoprotein cholesterol (LDL–C), and total bile acids (TBAs) kits were purchased from Nanjing Jiancheng Biotechnology Research Institute (Nanjing, China). The specific commercial testing kit numbers used were as follows: TC (A111-1-1), TG (A110-1-1), HDL-C (A112-1-1), LDL-C (A113-1-1), and TBAs (E003-2-1). Trizol Reagent and Reverse Transcription Kit were obtained from Tiangen Biochemical Technology (Beijing, China). TB Green^®^ Premix Ex TaqTM II (Tli RNaseH Plus) was provided by Takara Biomedical Technology (Shiga, Japan).

### 2.2. Animals and Experimental Design

Nine-week-old db/db diabetic mice (female, *n* = 10) and the same number of wild-type (wt) littermates of the same age were purchased from Cyagen Biosciences (Gu’an) Co., Ltd. (Guangzhou, China). All mice were housed in cages with an ambient temperature of 22 ± 2 °C, a relative humidity of 55 ± 5%, and a light/dark cycle of 12 h; feed and water were freely available.

The mice were divided into two groups after one week of adaptive feeding. The control group (CON, n = 10) consisted of wt mice with an average body weight of 19.34 ± 0.91 g and a blood glucose level of 7.35 ± 0.67 mmol/L, and the diabetes model group (db/db, *n* = 10) consisted of db/db mice with an average body weight of 50.80 ± 2.16 g and blood glucose level of 19.62 ± 2.04 mmol/L. The mice in both groups were fed an AIN93G diet [18] for 8 weeks, which was purchased from Trophic Animal Feed High-Tech Co., Ltd. (Nantong, China). This study was approved by the Ethical Committee for Animal Experimentation of the Academy of National Food and Strategic Reserves Administration, with the reference number (No. SYXK (Jing) 2019-0015).

### 2.3. Animal Sample Collection and Processing

Fasting blood glucose (FBG) was measured, and the mice were weighed once a week. The electronic blood glucose meter (Yuyue Medical Equipmeng, Danyang, China) was used to measure the blood glucose of mice. After a 12 h fast at the end of the eighth week of the experiment, blood from the eyeballs of the mice was collected. Serum was separated by centrifugation at 3000× *g* for 15 min at 4 °C, and stored at −20 °C for the serum index test. The liver, colon tissues and colonic contents were collected and stored at −80 °C for further analyses.

### 2.4. Serum Biochemical Analysis

Serum concentrations of TC, TG, HDL–C, LDL–C, and TBAs were measured by the colorimetric method using commercially available assay kits. The corresponding concentrations were obtained according to the formula between absorbance and detection index concentration. All reagent kits are based on the microplate method.

### 2.5. Quantitation of Bile Acids’ Composition in Colon Contents

The colon contents from both CON and db/db mice were sent to Majorbio Biotech (Shanghai, China) Co., Ltd., for BAs-targeted metabolomics analysis, as described by Yan et al. [19]. Then, 25 mg of the samples was weighted and homogenized with 380 µL of methanol/water (*v*/*v* = 4:1) mixed solvent containing 20 µL internal standard working solution (200 ng/mL). The homogenate was then ground by freezing the grinder for 6 min (−10 °C, 50 Hz), and sonicated at a low temperature for 30 min (5 °C, 40 KHz); then, it was stored at −20 °C for 30 min, and centrifuged at 4 °C for 15 min at 13,000 rcf. The BAs’ concentrations in the supernatants (200 µL) were measured by a ExionLC AD system coupled with an SCIEX QTRAP 6500+ Mass Spectrometer (Sciex, Framingham, MA, USA) with an ESI source. Chromatographic separations were operated with a Waters BEH C18 (150 × 2.1 mm, 1.7 µm). The LC-MS raw data were obtained using AB SCIEX quantitative software OS (Framingham, MA, USA) to obtain calibration equations. The BAs’ concentration of each sample was calculated according to the linear regression standard curve. 

### 2.6. Real-Time Quantitative PCR to Measure Level of BAs-Related Genes

The impact of T2D on critical genes related to BAs synthesis, transports and receptors was assessed. Total RNA of liver and colon tissues was extracted using Trizol Reagent, and 1 µg RNA was reversely transcribed into cDNA using the PrimeScript RT reagent kit with a gDNA Eraser according to the manufacturer’s instructions. RT-qPCR was performed with gene-specific primers (Table 1) and TB Green master mix on a CFX96 Real-time PCR detection system (Bio-Rad, Hercules, CA, USA). Primers were purchased from Sangon Biotech, and the efficiency of each primer was experimentally verified to be in the range of 90–110%. β-actin was used as an internal control to normalize the expression of the selected genes and the relative expression levels of these target genes were calculated by using the 2^−∆∆Ct^ method, as previously described [20].

### 2.7. Gut Microbiota Analysis

Total microbial genomic DNA in the colonic contents was extracted using a DNA isolation kit (Omega Bio-Tek, Norcross, GA, USA). The DNA extract was checked on 1% agarose gel, and DNA concentration and purity were determined with a NanoDrop2000 spectrophotometer (Thermo Scientific, Waltham, MA, USA). The V3-V4 hypervariable region of the 16S rRNA gene from the colon bacteria was selected for amplification using 341F (5′-ACTCCTACGGGAGGCAGCAG-3′) and 860R (5′-GGACTACHVGGGTWTCTAAT-3′) primers and sequenced by the Majorbio platform (Shanghai, China). PCR amplification was performed as follows: initial denaturation at 95 °C for 3 min, followed by 27 cycles of denaturing at 95 °C for 30 s, annealing at 60 °C for 30 s and extension at 72 °C for 45 s, and a single extension at 72 °C for 10 min, ending at 4 °C (T100 Thermal Cycler PCR thermocycler, BIO-RAD, Hercules, CA, USA). After electrophoresis, the PCR products were purified using the AMPure^®^ PB beads (Pacifc Biosciences, Menlo Park, CA, USA) and quantified with Qubit 4.0 (Thermo Fisher Scientific, Waltham, MA, USA). All data were analyzed on the Majorbio Cloud platform: https://cloud.majorbio.com (accessed on 10 November 2024). Raw FASTQ files were merged and demultiplexed, and quality control implementation was conducted. α diversity indices including the Chao index and Shannon index were calculated using Mothur v1.30.1, as previously described [21]. The Bray–Curtis method was used to perform principal component analysis to determine the similarity of microbial communities in the samples, identify the significant abundance of bacterial taxa between groups, and count the community composition of each sample at different species classification levels. A linear discriminant analysis (LDA) of effect size (LEfSe) was performed to identify the significantly abundant taxa (phyla, families, and genera) of bacteria among the different groups (LDA score > 4, *p* < 0.05).

### 2.8. Correlation Analysis

The correlation analysis included BAs and colon bacterial communities, and BAs with BAs-related genes and phenotypes were analyzed by calculating Spearman’s correlation coefficients. The correlation heatmap was plotted by https://www.bioinformatics.com.cn (last accessed on 10 December 2024), an online platform for data analysis and visualization, as previously described [22].

### 2.9. Statistical Analysis

The statistical analysis of this trial was performed using SPSS version 22.0 (SPSS Inc, Chicago, IL, USA) and each replicate served as an individual experimental unit. All data were presented as mean ± standard deviation (SD). To identify significant differences between the two groups, the *t*-test was utilized as the statistical analysis method. *p* values < 0.05 were considered significant.

## 3. Results

### 3.1. Body Weight, Fasting Blood Glucose and Serum Lipid Indices

The changes in body weight (BW) and fasting blood glucose (FBG) of the mice observed during the feeding period are shown in Table 2. The body weight of the db/db group mice was significantly higher than that of the CON group (*p* < 0.001). At week 8, the BW of mice in the CON group increased by 7.85%, while that of the db/db group decreased by 3.79%. In addition, compared with the CON group, the FBG levels were significantly increased in the db/db group (*p* < 0.001). Serum lipid levels are shown in Table 3. The concentrations of TC, TG, LDL–C, HDL–C, and TBA of db/db mice were significantly increased compared to those of the wild-type mice (*p* < 0.05). These results indicated typical features of db/db diabetic mice.

### 3.2. Alterations in Colonic Bile Acids Metabolism Between db/db and Wild-Type Mice

**Figure 1:**
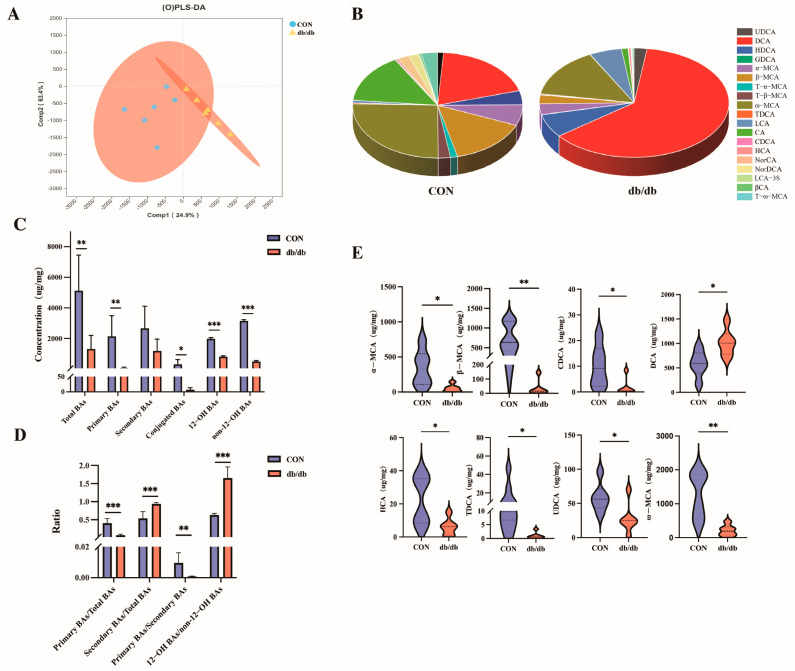
Distribution and composition of colonic BAs. (**A**): (O)PLS-DA score of colonic BAs profiles; the red area represents the 95% confidence intervals for the different groups. (**B**): Composition of colonic BAs profiles. (**C**): The concentrations of total BAs, primary BAs, secondary BAs, conjugated BAs, 12–OH BAs, and non-12–OH BAs. (**D**): The ratio of primary BAs to total BAs, secondary BAs to total BAs, primary BAs to secondary BAs, and 12–OH BAs to non-12–OH BAs. (**E**): Violin plots of differentially abundant colonic BAs, with dashed lines representing the 25th percentile, median, and 75th percentile, respectively. The individual graphics are shown in Supplementary Figure S1. Data are shown as mean ± SD (*n* = 6), compared with CON; * *p* < 0.05, ** *p* < 0.01, *** *p* < 0.001. UDCA, Ursodeoxycholic acid; DCA, Deoxycholic acid; HDCA, Hyodeoxycholic acid; GDCA, Glycodeoxycholic acid; α–MCA, α–Muricholic acid; β–MCA, β–Muricholic acid; T–α–MCA, Tauro–α–muricholic acid; T–β–MCA, Tauro–β–muricholic acid; ω–MCA, ω–Muricholic acid; TDCA, Tauroursodeoxycholic acid; LCA, Lithocholic acid; CA, Cholic acid; CDCA, Chenodeoxycholic acid; HCA, Hyocholic acid; NorCA, Norcholic acid; NorDCA, Nordehydrocholic acid; LCA–3S, Lithocholic acid 3–sulfate; βCA, β–Cholic acid; T–ω–MCA, Tauro–ω–muricholic acid.

The quantities and compositions of colonic BAs were determined by UPLC-MS. The Orthogonal Partial Least Squares Discriminant Analysis (OPLS-DA) model illustrates the difference in intestinal BAs’ composition between the db/db group and the CON group (Figure 1A). The CON group consisted mainly of ω–MCA (24.20%), DCA (18.46%), CA (14.37%), and β–MCA (14.27%), while the BAs’ composition of the db/db group was mainly DCA (60.19%), ω–MCA (14.12%), HDCA (6.65%), and LCA (5.34%) (Figure 1B). Overall, the concentrations of total BAs, primary BAs, conjugated BAs, 12 –OH BAs, and non-12–OH BAs were decreased (*p* < 0.05) in the db/db group compared with those in the CON group (Figure 1C). Meanwhile, significant changes in BAs’ ratios were observed, mainly in the form of a significant decrease in the ratio of primary to total BAs and the ratio of primary to secondary BAs (*p* < 0.01), while a significant increase in the ratios of secondary to total BAs and the ratio of 12–OH BA to non-12–OH BAs (*p* < 0.001) was observed (Figure 1D). Compared with the CON group, BAs’ analysis revealed that the levels of non-12–OH BAs were significantly decreased in the db/db group (*p* < 0.05), including α–MCA, β–MCA, CDCA, HCA, UDCA, ω–MCA and TDCA, while the level of 12–OH BAs (DCA) was significantly increased (*p* < 0.05) (Figure 1E). These results showed that the BAs’ profile in the intestinal tract was different between the db/db group and the CON group, suggesting that BAs may be the key factor causing metabolic disorders in diabetic model mice.

### 3.3. Changes in BAs-Related Genes Between db/db and Wild-Type Mice

**Figure 2:**
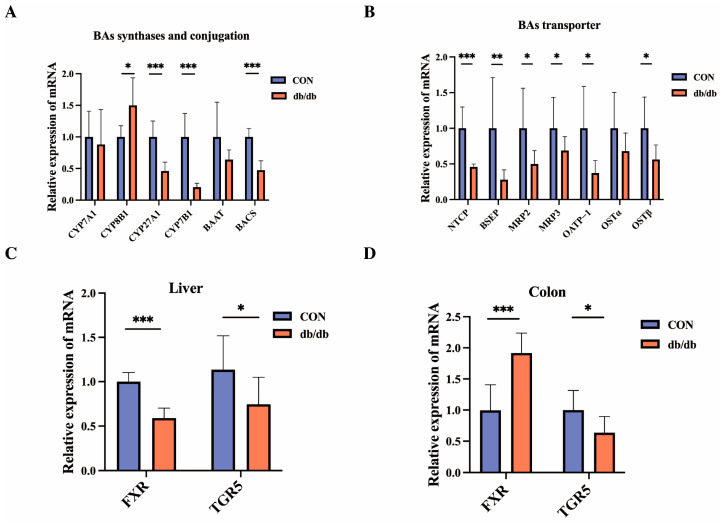
Analysis of gene expression influenced by different groups. (**A**): Relative mRNA expression of BAs critical enzymes and conjugation in the liver. (**B**): Relative mRNA expression of BAs transporter in the liver. (**C**): Relative mRNA expression of BAs receptor in the liver. (**D**): Relative mRNA expression of BAs receptor in the colon. Data are shown as mean ± SD (*n* = 8), compared with CON; * *p* < 0.05, ** *p* < 0.01, *** *p* < 0.001.

Based on the altered levels of BAs, we further examined the expression levels of BAs-related genes by qRT-PCR. Among the hepatic BAs synthases, the mRNA expressions of classical pathway synthetase CYP8B1 was significantly increased (*p* < 0.05), while the levels of alternative pathway synthetase CYP27A1 and CYP7B1 as well as conjugation gene BACS were significantly decreased in the db/db group compared with the CON group (*p* < 0.001) (Figure 2A). Among the hepatic BAs’ transporters, the mRNA expression levels of NTCP, BSEP, MRP2, MRP3, OATP–1 and OSTβ were decreased in the db/db group relative to those of the CON group (*p* < 0.05) (Figure 2B). Moreover, the expression levels of BAs receptor FXR and TGR5 (*p* < 0.05) in the liver were significantly decreased in the db/db mice compared with the wild-type mice (Figure 2C). In the colon, the FXR expression level of db/db mice was markedly higher than that of CON mice (*p* < 0.05), while the TGR5 level showed a significant down-regulation in the db/db group (*p* < 0.05) (Figure 2D). These results suggest that db/db diabetic mice altered the expression levels of liver BAs synthesis and transport, as well as BAs’ receptor in the liver and intestine.

### 3.4. Microbiota Profile of the Colon Between db/db and Wild-Type Mice

**Figure 3:**
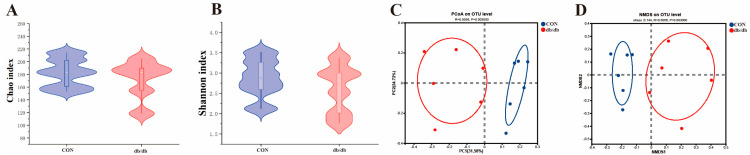
Analysis of intestinal microbiota diversity in different groups. (**A**): Chao index of intestinal bacteria. (**B**): Shannon index diversity index of intestinal bacteria. (**C**): PCoA analysis result of intestinal bacteria. (**D**): NMDS analysis result of intestinal bacteria. The white dots in the violin chart represent the median. Different colored circles represent different groups in diversity analysis.

In this study, 16S rDNA sequencing, combined with bioinformatics methods, was used to analyze the composition of colonic gut microbes in mice. The α-diversity analysis of intestinal microorganisms in mice showed that the observed Chao and Shannon index levels in the db/db group were similar to those in the CON group (*p* > 0.05) (Figure 3A,B). However, the data from principal component analysis (PCoA) and non-metric multi-dimensional scaling (NMDS) based on β-diversity showed that the structure of the intestinal flora in the db/db group significantly differed from that in the CON group (R = 0.506, *p* = 0.003) (Figure 3C,D).

**Figure 4:**
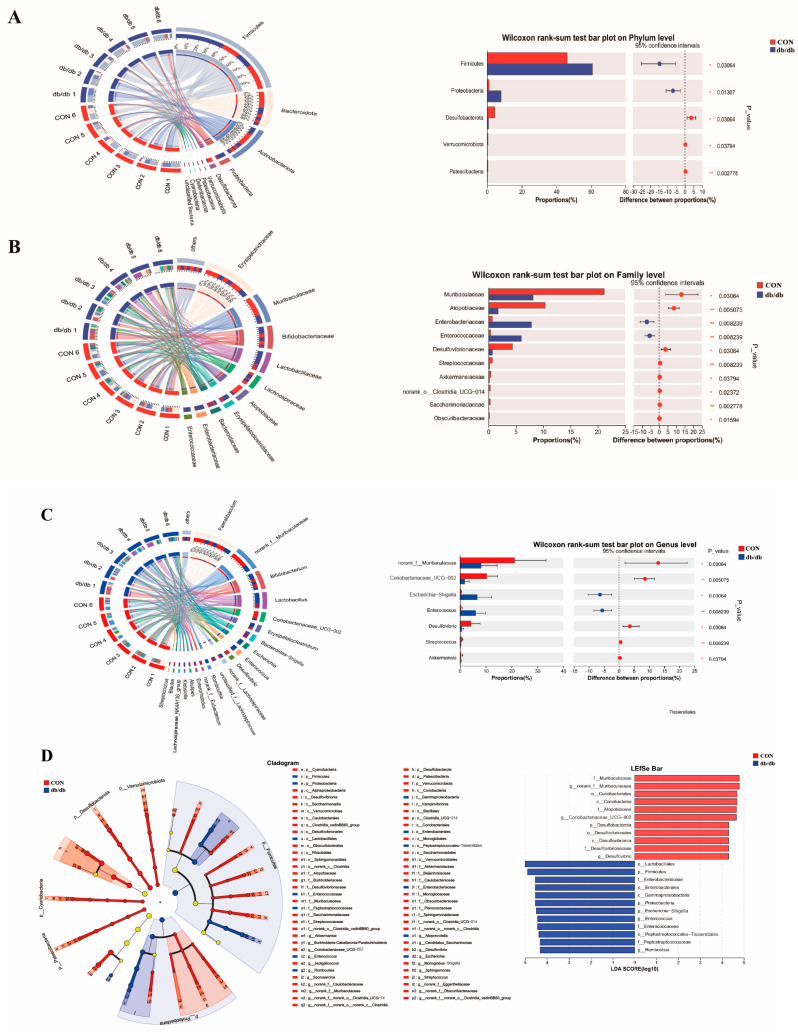
Analysis of the structure of the intestinal microbiota. The abundance of the intestinal microbiota composition at the phylum level (**A**), family level (**B**) and genus level (**C**). (**D**): LEfSe analysis results of intestinal microbiota at the level of OTUs. Data are shown as mean ± SD (*n* = 6), compared with CON; * *p* < 0.05, ** *p* < 0.01.

To further determine the change in the gut microbiota structure of mice with T2D, the colonic bacterial composition of the db/db and CON groups was compared at the phylum, family, and genus levels. At the phylum level, db/db mice showed a significant reduction in the relative abundance of *Desulfobacterota* and an increase in the relative abundances of *Firmicutes* and *Proteobacteria* as compared to the CON mice (Figure 4A). At the family level, the abundances of *Desulfovibrio*, *norank_f_Muribaculaceae*, and *Coriobacteriaceae_UCG–002* of db/db mice were significantly reduced, while the abundances of *Enterobacteriaceae* and *Enterococcaceae* were increased relative to those of CON mice (Figure 4B). At the genus level, the db/db group had significantly higher abundances of *Escherichia–Shigella* and *Enterococcus,* and lower abundances of *Desulfovibrio*, *norank_f_Muribaculaceae*, *Akkermansia*, *Coriobacteriaceae_UCG–002*, and *Streptococcus* than the CON group (Figure 4C). The LEfSe results showed that the colon of mice in the db/db group had enriched *Firmicutes*, *Proteobacteria*, *Enterobacteriaceae*, *Enterococcaceae*, *Peptostreptococcaceae, Escherichia–Shigella*, *Enterococcus*, and *Romboutsia*, while the CON group had enriched *Desulfobacterota*, *Muribaculaceae*, *Atopobiaceae*, *Desulfovibrionaceae*, *norank_f_Muribaculaceae*, *Coriobacteriaceae_UCG–002*, and *Desulfovibrio* (Figure 4D). The source data of the gut microbiota are shown in Supplementary Table S1. These results revealed an obvious alteration in the pattern of the gut microbiota in db/db mice.

### 3.5. Association Analysis of Bile Acids, Gut Microbiota, and Metabolic Indicators

**Figure 5:**
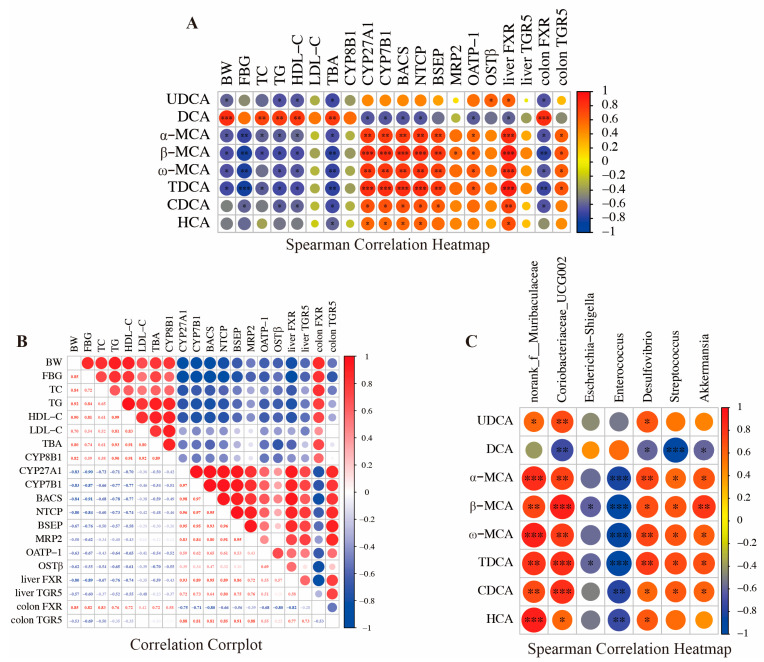
Spearman’s correlation analysis for association analysis between BAs and metabolic indicators intensities (**A**). Intra-group correlation corrplot analysis of metabolic indicators (**B**). Heat maps of the spearman rank correlation coefficient and significant tests between the BAs and differential bacteria (**C**). Comparison with CON. The color intensity (red and blue) and size of the circle indicate the strength of the correlation depicted as an r–value. Asterisks on each circle indicate the *p*–value; *** ≤ 0.001, ** ≤ 0.01 and * ≤ 0.05. The analysis was performed using the corrplot package in R (v4.1).

The levels of CDCA, HCA, α–MCA, β–MCA, UDCA, TDCA, and ω–MCA exhibited an inverse relationship with BW, FBG, TC, TG, HDL–C, TBA, and colon FXR (Figure 5A), while a positive correlation with the expression of CYP27A1, CYP7B1, BACS, NTCP, BSEP, MRP2, OATP–1, and OSTβ was demonstrated. In contrast, DCA displayed a contrary trend, showing a positive correlation with BW, TC, TG, HDL–C, TBA, and colon FXR, while it was negatively associated with OSTβ. Furthermore, the results of the intra-group correlation analysis revealed a positive correlation among different phenotype groups (Figure 5B). Except for colon FXR, the expression levels of various genes also showed a positive correlation. In contrast, there was a negative correlation between different phenotypes and gene expression levels. In addition, Spearman’s rank correlations coefficient and significance tests revealed a correlation between the BAs and various bacteria (Figure 5C). The levels of CDCA, HCA, α–MCA, β–MCA, UDCA, TDCA, and ω–MCA were positively correlated with the relative abundance of *norank_f__Muribaculaceae*, *Coriobacteriaceae_UCG–002, Desulfovibrio*, *Streptococcus* and *Akkermansia*, while they were negatively correlated with the relative abundance of *Enterococcus and Escherichia–Shigella*. Furthermore, DCA concentration was significantly negatively correlated with the abundance of *Coriobacteriaceae_UCG–002*, *Desulfovibrio*, *Streptococcus* and *Akkermansia.*

## 4. Discussion

Type 2 diabetes (T2D), a chronic metabolic disorder that accounts for more than 90% of all diabetes cases, can lead to severe complications in multiple organs and has emerged as a significant threat to global health [23]. Bile acids (BAs) play an important role in the regulation of host metabolic homeostasis [24]. Numerous studies have shown the disruption of BAs metabolism and intestinal flora in diabetic populations and animal models. However, systematic studies on intestinal BAs’ profile, BAs’ metabolism regulation and intestinal microbiota in T2D are still insufficient. db/db mice are leptin receptor dysfunction mice that exhibit severe obesity and hyperglycemia, making them common animal models for T2D.

In the current study, we found that db/db mice had significantly higher blood glucose and serum lipids than wild-type mice, aligned with previous research findings [25]. Emerging evidence reported that BAs’ metabolism plays a causal role in the pathogenesis of T2D. However, the precise mechanisms through which the endogenous BAs’ profile regulates homeostasis remain incompletely defined. We observed a decrease in the concentrations of colonic total BAs and non-12 OH–BAs, while an increase in the levels of 12–OH BAs was observed in db/db mice. These non-12–OH BAs were predicted to be positively associated with body weight, fasting blood glucose and lipid levels (Figure 5A). Ruan et al. (2022) confirmed that the reduction in intestinal total BAs was related to the disturbance of enterohepatic circulation and BAs’ reabsorption disorder, and was closely related to metabolic abnormalities [26]. The increased 12–OH BAs/non-12–OH BAs ratio was associated with lower insulin sensitivity and a higher plasma TG level [27]. This may mean 12–OH BAs and non-12–OH BAs affect the rate of BAs’ enterohepatic circulation and the regulation of metabolism to different degrees. For example, non-12–OH BAs could accelerate the circulation and excretion of BAs, thereby reducing the level of circulating cholesterol metabolism and regulating lipid metabolism balance [28]. Additionally, we found that db/db mice decreased the ratio of primary BAs to secondary BAs, while they increased the ratio of secondary BAs to total BAs. Consistent with our findings, Armin et al. (2020) also found that T2D mice had lower levels of primary BAs and higher levels of secondary BAs in the intestine compared to healthy mice [29]. A decreased ratio of primary BAs to secondary BAs and an increased secondary BAs level were associated with the induction of metabolic diseases, such as high blood glucose and lipid accumulation [30,31]. However, some of the studies did not elaborate on these BAs results, which may experimentally take place in different animal models and feeding conditions. In the db/db mice, we found DCA was the most prevalent BA component, and its level was significantly higher than that of wild mice, and showed a positive correlation with metabolic disorder indicators (Figure 5A). These results are similar to those of Karolina et al. (2018), who found DCA was associated with an impaired glucose metabolism and worsened lipid profile in T2D patients [32]. Dietary supplementation of DCA disrupted hepatic endoplasmic reticulum homeostasis in mice, which impaired systemic glucose regulation [33]. Taken together, these observations suggested that db/db mice might induce metabolic disorders by decreasing total BAs’ and non-12–OH BAs’ levels, as well as the ratios of primary to secondary BAs, and increase the level of secondary BAs, DCA, in the colon.

The dysregulation of BAs’ synthesis and transport, as well as receptors’ expression, was also associated with metabolic disorders, such as diabetes, obesity and cardiovascular diseases. In our study, db/db mice exhibited an increase in the expression of CYP8B1, while decreased CYP27A1 and CYP7B1 levels in the liver were observed. These results suggest the conversion of BAs synthesis from the alternative pathway to the classical pathway. The activation of BAs’ alternative pathway plays an important role in the treatment of metabolic diseases such as hyperglycemia and hyperlipidemia [6]. CYP8B1 and CYP7B1 activities are key factors determining the homeostasis of glucose and lipid metabolism. Consistent with our findings, the expression of CYP8B1 was elevated in diabetic and obese mice liver [34,35]. CYP8B1 is a key enzyme in classical pathway synthesis, and the overexpression of it elevated 12–OH BA levels and induced lipogenesis gene expression [36]. The alternative pathway is initiated by CYP27A1 and further hydroxylated through CYP7B1 to produce non-12–OH BAs [6]. It has been reported that the decreased expression of liver CYP7B1 is related to the disorder of glucose metabolism [15]. After the synthesis of BAs in the liver, conjugated BAs are formed under the action of BAAT and BACS. Reduced BACS and conjugated BAs levels were found in this study. BACS have been shown to be direct targets of BAs’ receptor in human and murine hepatocytes to regulate BAs and lipid homeostasis [37].

Moreover, the db/db mice inhibited the expression of BAs transporters, which also exhibited a positive correlation with non-12–OH BAs levels and negatively correlated with the glycolipid metabolic phenotype (Figure 5B,C). Reduced BAs’ transporters indicated a reduction in the extent to which BAs in the ileum enter the epithelial cells, pass through the portal circulation, and flow back to the hepatocytes, which disrupts the hepatic and intestinal circulation of BAs [8]. These results may explain the decrease in total BAs’ and non-12–OH BAs’ levels. BAs can change the host’s metabolism by binding to nuclear receptors, including FXR and TGR5. The db/db mice suppressed the mRNA levels of FXR and TGR5 in the liver, while they decreased the level of TGR5 with an increase in FXR in the colon. We also predicted that TGR5 in the colon and FXR in the liver were negatively correlated with blood glucose and lipid levels, whereas FXR in the colon was the opposite of the phenomenon above (Figure 5C). Studies have shown that FXR activation in the liver could inhibit the synthesis of BAs’ classical pathway through the small heterodimer partner pathway, which has a beneficial effect on metabolic homeostasis [38]. TGR5’s activation promoted energy expenditure and induced glucagon-like peptide–1 (GLP–1) release to regulate blood glucose homeostasis and reduce obesity [39]. Suppression of FXR expressed in the intestinal activates CYP7B1 level via the up–regulating fibroblast growth factor 19/15 [40]. In contrast to TGR5, the inhibition of FXR in intestinal L cells induces GLP–1 synthesis to promote better glucose homeostasis [41]. These findings indicated that db/db mice might induce abnormal glucose and lipid metabolism by promoting the transformation of BAs’ alternative synthesis pathway into the classical pathway and suppressing the expression of BAs’ transporters and receptors. However, previous studies of BAs’ receptors have not included the liver X–activated receptor and pregnane X receptor, which play key roles in metabolism, and these may need to be reported in future studies.

A growing body of evidence suggests that BAs are increasingly appreciated to be critically involved in the regulation of metabolism through interacting with gut microbiota [42]. Wang et al. (2022) found that fecal microbiota transplantation in diabetic patients interferes with glucose metabolism, and the possible mechanism is that the intestinal flora’s structure alters the ability of intestinal flora to metabolize BAs, increases the ratio of 12–OH BAs to non-12–OH BAs, and affects the BAs/GLP–1 pathway [43]. This result revealed that gut microbiota may regulate the ratio of 12–OH BAs to non-12–OH BAs and induced hepatic synthesis of BAs with hydroxyl groups at different moieties. We observed that db/db mice decreased the abundances of *Desulfovibrio*, *norank_f_Muribaculaceae*, *Akkermansia* and *Coriobacteriaceae_UCG–002*, which showed a strong positive correlation with non-12–OH BAs, and negatively correlated with DCA (Figure 5C). Accordingly, we found that the relative abundance of *parabacterioids distason* was increased but did not reach a significant level. Wang et al. (2024) also observed that an increased ratio of non-12–OH BAs to 12–OH BAs was significantly positively correlated with *Akkermansia* [44]. A similar study has also shown that 12–OH BAs, secondary BAs and DCA were negatively correlated with *Akkermansia* [45]. In addition, Li et al. (2022) found a decrease in *Parabacteroides distasonis* in calorie-restricted mice, which was positively correlated with the proportion of non–12–OH BAs and glucose and lipid metabolism homeostasis [46]. Furthermore, increased abundances of *Escherichia–Shigella* and *Enterococcus*, which had a positive correlation with DCA, were observed in the present study. DCA is a secondary BA that is converted from primary BA by 7α–HSDH- and 7β–HSDH-producing bacteria such as *Escherichia–Shigella* and *Clostridium sensu stricto 2* [47]. These results suggested that db/db mice might have decreased the proportion of non-12–OH BAs and increased the DCA level by inhibiting the abundance of *Akkermansia* and promoting the enrichment of *Escherichia–Shigella*. In future studies, based on the results of key bacteria, it will be possible to further analyze the direct role of each type of bacteria on BAs’ metabolism, and thus better explain its mechanistic changes.

## 5. Conclusions

In summary, we have demonstrated that db/db mice produce a significantly lower amount of non-12–OH BAs and a higher level of 12–OH BAs (DCA), and harbor fewer non-12–OH BAs–positive bacteria *Akkermansia*, *norank_f__Muribaculaceae*, *Coriobacteriaceae_UCG–002, Desulfovibrio*, and *Streptococcus*, with more abundance of secondary BAs-producing bacteria *Escherichia–Shigella* than wild-type littermates. These modified BAs activated the intestinal FXR receptor, inhibiting both alternative BA synthetic pathways, and receptors FXR and TGR5 in the liver, thereby inducing metabolic disorders. The strong link between BAs and glycolipid metabolism raises the possibility that modulating BAs’ metabolism might be a promising treatment strategy for T2D. However, this study is partially flawed. In subsequent studies, fecal microbiota transplantation of db/db mice will be conducted to clarify the direct regulatory role of intestinal flora on BAs’ metabolism, and further identify which species of the gut microbiota have a regulatory effect on T2D via BAs.

## Figures and Tables

**Table 1:** Primer sequences used for real-time PCR.

Gene	Forward Primer (5′-3′)	Reverse Primer (5′-3′)
**Bile acids synthesis**		
CYP7A1	GGGATGTATGCCTTCTGCT	AGTGCCGGAAATACTTGGT
CYP8B1	AGCTCCTGGTGTGAAGATG	GTCCTTGGTGTAGCCGAA
CYP27A1	ATCGGCACCTTTCCTGA	TTTTAGCAGAGGCATGTGG
CYP7B1	CTTTGCCGCCACCTTAC	TCCCCACAAGGAAGACAG
BAAT	TGCAGAGCAAGCACAGAACATC	CTGTTTCAGGTGACCCACTCCA
BACS	TCCCGAAGCCAGCCATCCTC	GATCCCAACGACAAGTCCCATCAC
**Bile acids transports**		
NTCP	TCCTCCCTGATGCCTTT	AATCCTGTTTCCATGCTGA-
BSEP	CCGCCAGCATCTTTGAGACA	CAATTTCACCCT TAATTCGATCCAG
MRP2	GCACCGACTATCCAGCATCT	AACCAAAGGCACTCCAGAAA
MRP3	CACACCACAACCACCTTCAC	TCAGGGTAGAGTCCAATGAGC
OATP-1	TCCCCGCAGTCTTCATTCTAA	TGGATGTCGCCAGGGAAAT
OSTα	GTTCGCCTCCCTATTCCTCT	ACCTTGTGGTCTTTCCTTCG
OSTβ	GTATTTTCGTGCAGAAGATGCG	TTTCTGTTTGCCAGGATGCTC
**Bile acids receptors**		
FXR	ACCAGGGAGAGACTGAGGT	AGGTGAGCGCGTTGTAGT
TGR5	GCATGGGGAACGCTACA	AGGCTGGCAAAGAACAGG

**Note:** Bold type indicates function of related genes.

**Table 2:** Body weight and fasting blood glucose levels between CON and db/db groups.

Week	Body Weight (g)			Fasting Blood Glucose (mmol/L)		
	CON	db/db	*p* Value	CON	db/db	*p* Value
1	20.51 ± 1.00	49.58 ± 3.73	<0.001	7.93 ± 0.72	21.01 ± 4.47	<0.001
2	20.89 ± 1.14	49.72 ± 4.93	<0.001	6.11 ± 0.73	22.17 ± 5.60	<0.001
3	20.86 ± 0.69	49.33 ± 5.79	<0.001	6.75 ± 0.68	24.80 ± 5.72	<0.001
4	21.48 ± 1.03	50.22 ± 5.01	<0.001	7.15 ± 0.62	23.76 ± 4.03	<0.001
5	21.39 ± 0.95	48.97 ± 5.59	<0.001	7.18 ± 0.59	22.37 ± 6.06	<0.001
6	22.80 ± 1.18	48.10 ± 5.73	<0.001	7.35 ± 0.47	23.81 ± 5.26	<0.001
7	23.25 ± 1.93	48.56 ± 5.71	<0.001	7.54 ± 0.63	25.76 ± 4.82	<0.001
8	22.12 ± 1.28	47.7 ± 5.30	<0.001	7.08 ± 0.51	22.11 ± 3.92	<0.001

Data are shown as mean ± SD (*n* = 10).

**Table 3:** Serum lipid indices between CON and db/db groups.

	CON	db/db	*p* Value
TC	6.75 ± 0.87	12.87 ± 3.27	0.0018
TG	0.81 ± 0.098	1.41 ± 0.22	<0.001
LDL–C	2.08 ± 0.63	3.60 ± 1.24	0.026
HDL–C	1.03 ± 0.15	2.71 ± 0.77	<0.001
TBA	10.31 ± 2.32	15.06 ± 2.59	0.0014

Data are shown as mean ± SD (*n* = 10).

## Data Availability

The data that support the findings of this study are available in this article or from the corresponding author upon reasonable request.

## Supplementary Materials

The following supporting information can be downloaded at: https://www.mdpi.com/article/10.3390/biology14030291/s1, Supplementary Figure S1: Individual graphics in the main text. Supplementary Table S1: Relative abundance of intestinal flora at the phylum, family, genus and OTU levels of different mice, *n* = 6.
