# Supplementary material for: Imbalance of Bile Acids Metabolism Mediated by Gut Microbiota Contributed to Metabolic Disorders in Diabetic Model Mice
Source: Biology (Basel). 2025 Mar 13;14(3):291. doi: 10.3390/biology14030291 (PMC11940414; doi:10.3390/biology14030291)
Supplement: Supplementary file 1 [file biology-14-00291-s001.zip › Supplementary Files/Supplementary Figure S1.pdf]

Supplementary Figure S1：Individual graphics in the main text

1A

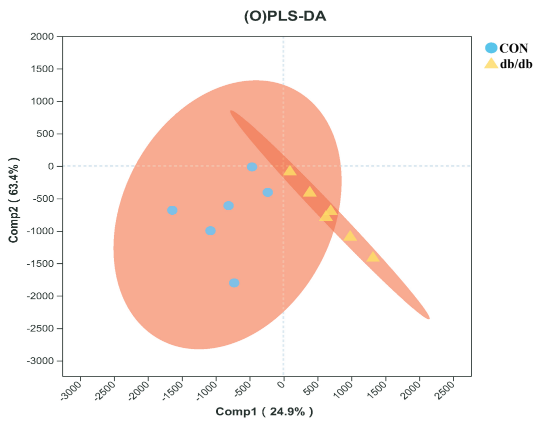

1B

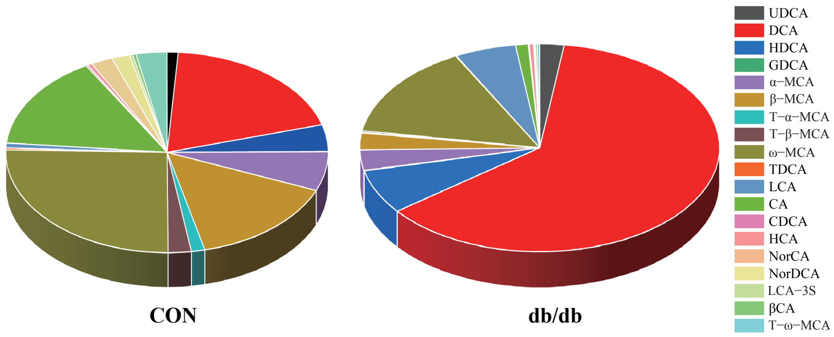

1C

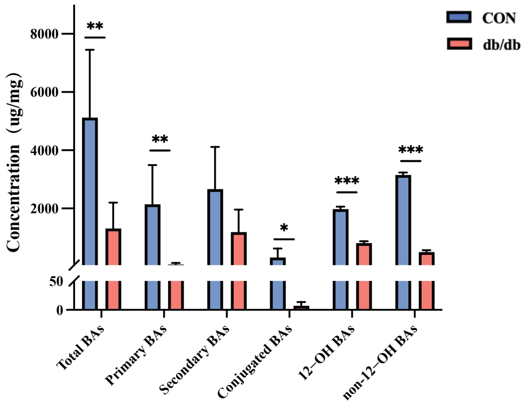

1D

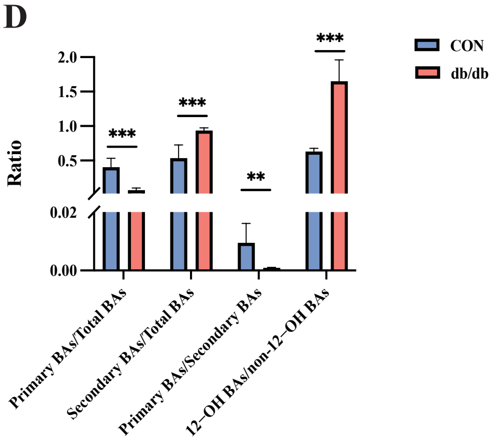
CON
db/db
Ratio
2.0
1.5
1.0
0.5
0.02
0.00
***
***
**
***
Primary BAs/Total BAs
Secondary BAs/Total BAs
Primary BAs/Secondary BAs
12−OH BAs/non-12−OH BAs

1E

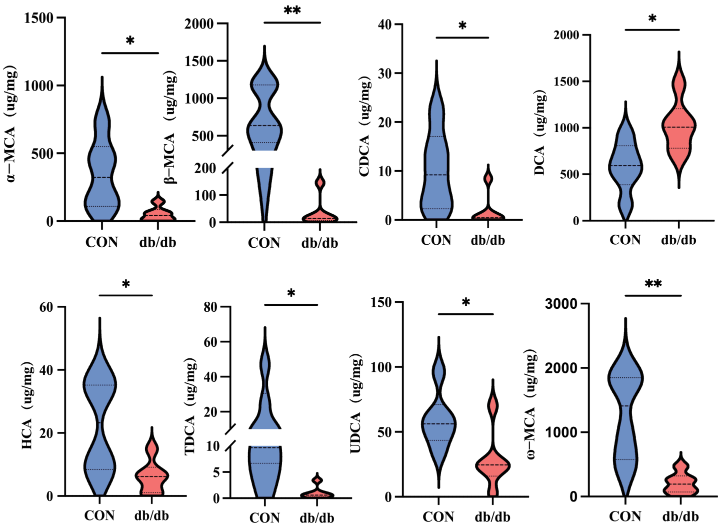
α−MCA (ug/mg)
β−MCA (ug/mg)
CDCA (ug/mg)
DCA (ug/mg)
HCA (ug/mg)
TDCA (ug/mg)
UDCA (ug/mg)
ω−MCA (ug/mg)
CON
db/db
*
**

2A

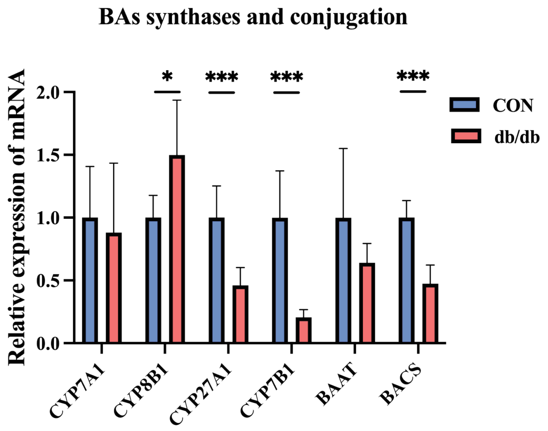
BAs synthases and conjugation
Relative expression of mRNA
2.0
1.5
1.0
0.5
0.0
*
***
***
***
CON
db/db
CYP7A1
CYP8B1
CYP27A1
CYP7B1
BAAT
BACS

2B

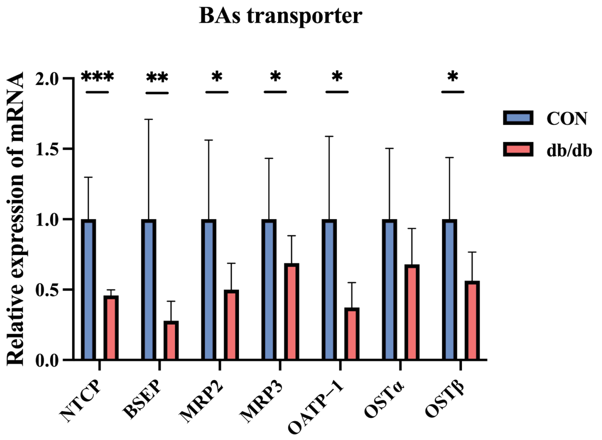
BAs transporter
Relative expression of mRNA
2.0
1.5
1.0
0.5
0.0
***
**
*
*
*
*
CON
db/db
NTCP
BSEP
MRP2
MRP3
OATP-1
OSTα
OSTβ

2C

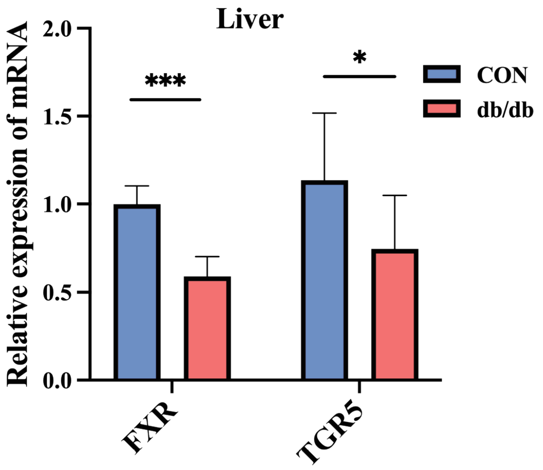
Liver
Relative expression of mRNA
2.0
1.5
1.0
0.5
0.0
***
*
CON
db/db
FXR
TGR5

2D

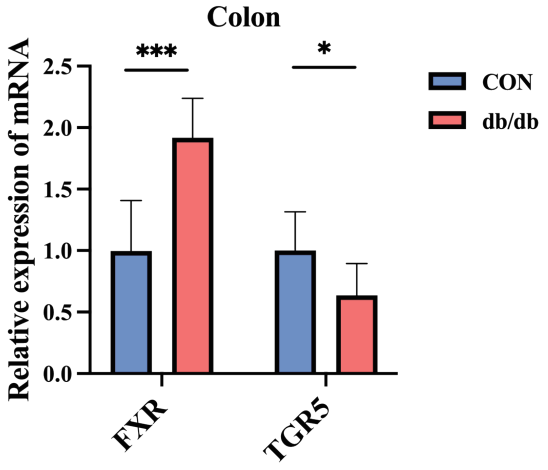
Colon
Relative expression of mRNA
2.5
2.0
1.5
1.0
0.5
0.0
***
*
CON
db/db
FXR
TGR5

3A

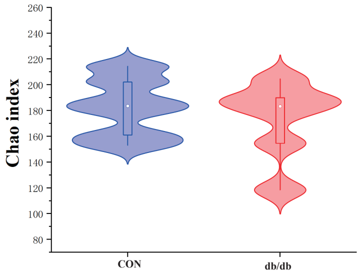
Chao index
260
240
220
200
180
160
140
120
100
80
CON
db/db

3B

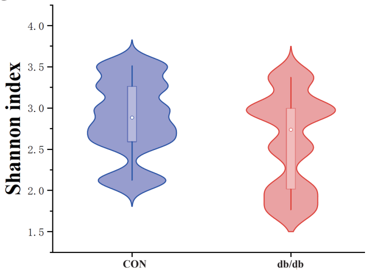
Shannon index
4.0
3.5
3.0
2.5
2.0
1.5
CON
db/db

3C

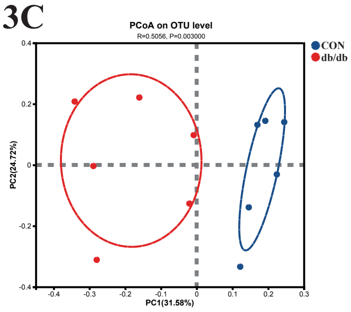
PCoA on OTU level
R=0.5056, P=0.003000
CON
db/db
PC2(24.72%)
PC1(31.58%)

3D

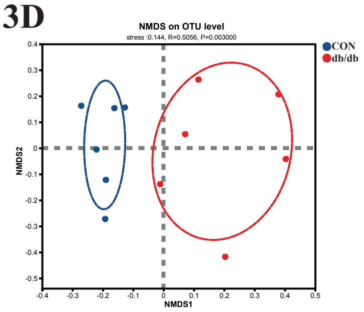
NMDS on OTU level
stress :0.144, R=0.5056, P=0.003000
CON
db/db
NMDS2
NMDS1

4A

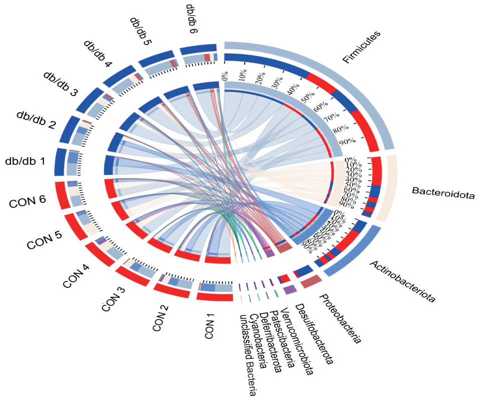
db/db 6
db/db 5
db/db 4
db/db 3
db/db 2
db/db 1
CON 6
CON 5
CON 4
CON 3
CON 2
CON 1
Firmicutes
Bacteroidota
Actinobacteriota
Proteobacteria
Desulfobacterota
Verrucomicrobiota
Patescibacteria
Deferribacterota
Cyanobacteria
unclassified Bacteria

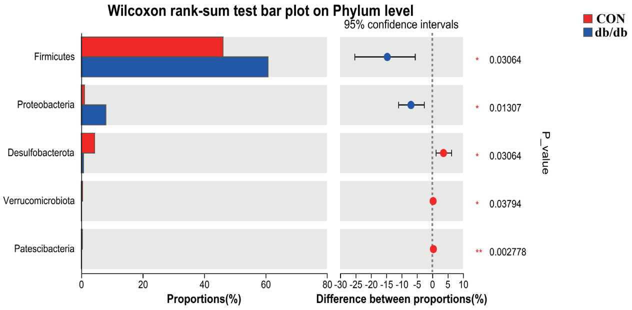
Wilcoxon rank-sum test bar plot on Phylum level
95% confidence intervals
CON
db/db
Firmicutes
Proteobacteria
Desulfobacterota
Verrucomicrobiota
Patescibacteria
0.03064
0.01307
0.03064
0.03794
0.002778
P_value
Proportions(%)
Difference between proportions(%)

4B

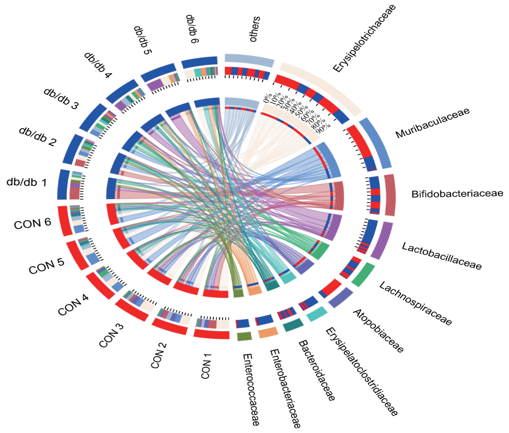

Erysipelotrichaceae
Muribaculaceae
Bifidobacteriaceae
Lactobacillaceae
Lachnospiraceae
Atopobiaceae
Erysipelatoclostridiaceae
Bacteroidaceae
Enterobacteriaceae
Enterococcaceae
others
db/db 6
db/db 5
db/db 4
db/db 3
db/db 2
db/db 1
CON 6
CON 5
CON 4
CON 3
CON 2
CON 1

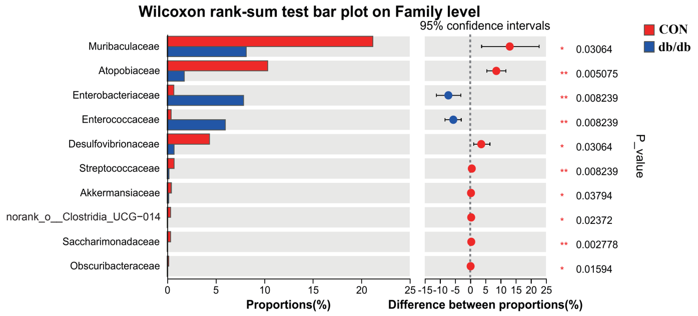

Wilcoxon rank-sum test bar plot on Family level
95% confidence intervals
CON
db/db
Muribaculaceae * 0.03064
Atopobiaceae ** 0.005075
Enterobacteriaceae ** 0.008239
Enterococcaceae ** 0.008239
Desulfovibrionaceae * 0.03064
Streptococcaceae ** 0.008239
Akkermansiaceae * 0.03794
norank_o__Clostridia_UCG-014 * 0.02372
Saccharimonadaceae ** 0.002778
Obscuribacteraceae * 0.01594
P_value
0 5 10 15 20 25
Proportions(%)
-15 -10 -5 0 5 10 15 20 25
Difference between proportions(%)

4C

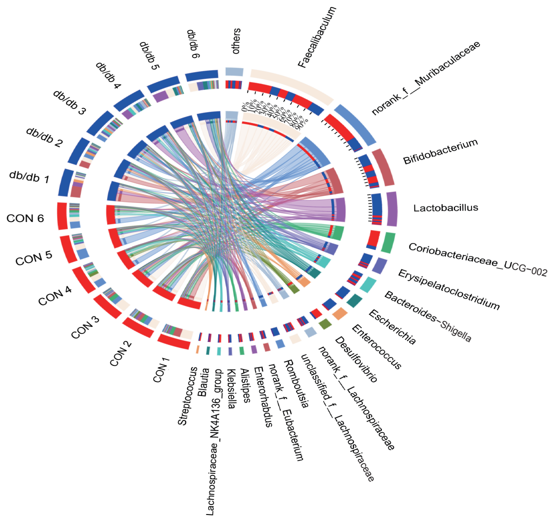

Faecalibaculum
norank_f__Muribaculaceae
Bifidobacterium
Lactobacillus
Coriobacteriaceae_UCG-002
Erysipelatoclostridium
Bacteroides
Escherichia-Shigella
Enterococcus
Desulfovibrio
norank_f__Lachnospiraceae
unclassified_f__Lachnospiraceae
Romboutsia
norank_f__Eubacterium
Enterorhabdus
Alistipes
Klebsiella
Lachnospiraceae_NK4A136_group
Blautia
Streptococcus
others
db/db 6
db/db 5
db/db 4
db/db 3
db/db 2
db/db 1
CON 6
CON 5
CON 4
CON 3
CON 2
CON 1

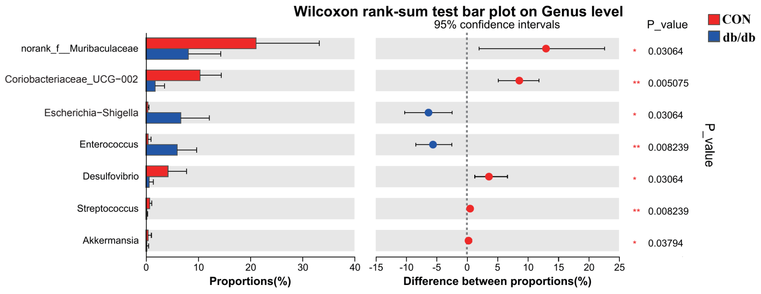

Wilcoxon rank-sum test bar plot on Genus level
95% confidence intervals
P_value
CON
db/db
norank_f__Muribaculaceae * 0.03064
Coriobacteriaceae_UCG-002 ** 0.005075
Escherichia-Shigella * 0.03064
Enterococcus ** 0.008239
Desulfovibrio * 0.03064
Streptococcus ** 0.008239
Akkermansia * 0.03794
0 10 20 30 40
Proportions(%)
-15 -10 -5 0 5 10 15 20 25
Difference between proportions(%)

4D

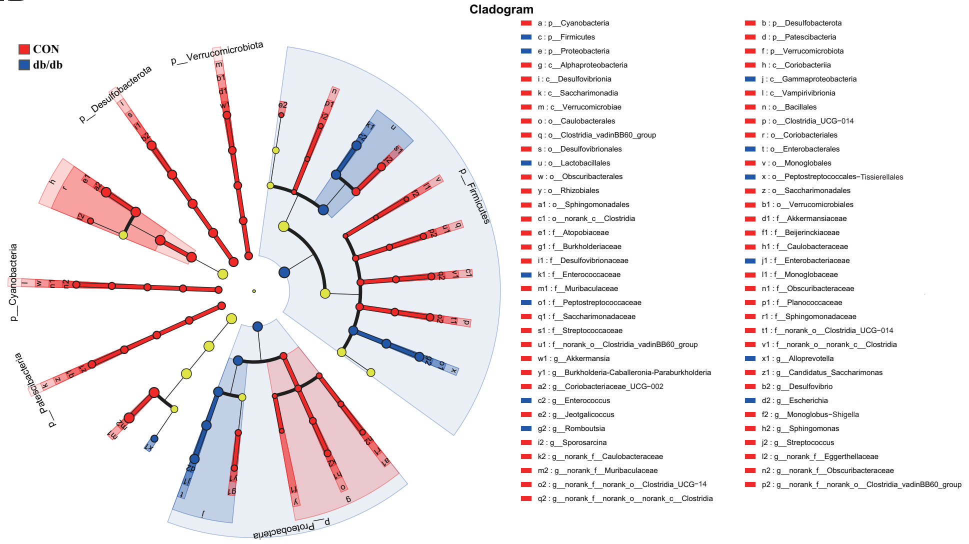

Cladogram
CON
db/db
p__Verrucomicrobiota
p__Desulfobacterota
p__Firmicutes
p__Cyanobacteria
p__Patescibacteria
p__Proteobacteria
a : p__Cyanobacteria
b : p__Desulfobacterota
c : p__Firmicutes
d : p__Patescibacteria
e : p__Proteobacteria
f : p__Verrucomicrobiota
g : c__Alphaproteobacteria
h : c__Coriobacteriia
i : c__Desulfovibrionia
j : c__Gammaproteobacteria
k : c__Saccharimonadia
l : c__Vampirivibrionia
m : c__Verrucomicrobiae
n : o__Bacillales
o : o__Caulobacterales
p : o__Clostridia_UCG-014
q : o__Clostridia_vadinBB60_group
r : o__Coriobacteriales
s : o__Desulfovibrionales
t : o__Enterobacterales
u : o__Lactobacillales
v : o__Monoglobales
w : o__Obscuribacterales
x : o__Peptostreptococcales-Tissierellales
y : o__Rhizobiales
z : o__Saccharimonadales
a1 : o__Sphingomonadales
b1 : o__Verrucomicrobiales
c1 : o__norank_c__Clostridia
d1 : f__Akkermansiaceae
e1 : f__Atopobiaceae
f1 : f__Beijerinckiaceae
g1 : f__Burkholderiaceae
h1 : f__Caulobacteraceae
i1 : f__Desulfovibrionaceae
j1 : f__Enterobacteriaceae
k1 : f__Enterococcaceae
l1 : f__Monoglobaceae
m1 : f__Muribaculaceae
n1 : f__Obscuribacteraceae
o1 : f__Peptostreptococcaceae
p1 : f__Planococcaceae
q1 : f__Saccharimonadaceae
r1 : f__Sphingomonadaceae
s1 : f__Streptococcaceae
t1 : f__norank_o__Clostridia_UCG-014
u1 : f__norank_o__Clostridia_vadinBB60_group
v1 : f__norank_o__norank_c__Clostridia
w1 : g__Akkermansia
x1 : g__Alloprevotella
y1 : g__Burkholderia-Caballeronia-Paraburkholderia
z1 : g__Candidatus_Saccharimonas
a2 : g__Coriobacteriaceae_UCG-002
b2 : g__Desulfovibrio
c2 : g__Enterococcus
d2 : g__Escherichia
e2 : g__Jeotgalicoccus
f2 : g__Monoglobus-Shigella
g2 : g__Romboutsia
h2 : g__Sphingomonas
i2 : g__Sporosarcina
j2 : g__Streptococcus
k2 : g__norank_f__Caulobacteraceae
l2 : g__norank_f__Eggerthellaceae
m2 : g__norank_f__Muribaculaceae
n2 : g__norank_f__Obscuribacteraceae
o2 : g__norank_f__norank_o__Clostridia_UCG-14
p2 : g__norank_f__norank_o__Clostridia_vadinBB60_group
q2 : g__norank_f__norank_o__norank_c__Clostridia

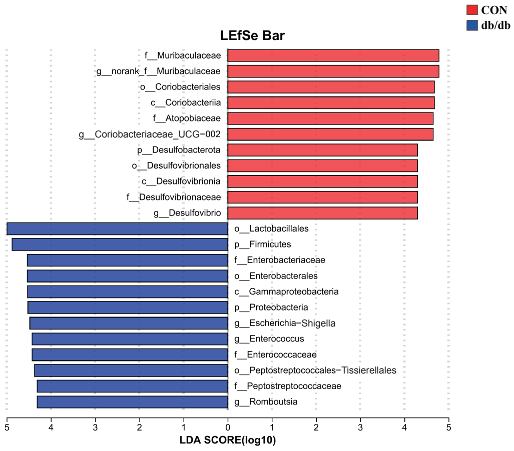

CON
db/db
LEfSe Bar
f__Muribaculaceae
g__norank_f__Muribaculaceae
o__Coriobacteriales
c__Coriobacteriia
f__Atopobiaceae
g__Coriobacteriaceae_UCG-002
p__Desulfobacterota
o__Desulfovibrionales
c__Desulfovibrionia
f__Desulfovibrionaceae
g__Desulfovibrio
o__Lactobacillales
p__Firmicutes
f__Enterobacteriaceae
o__Enterobacterales
c__Gammaproteobacteria
p__Proteobacteria
g__Escherichia-Shigella
g__Enterococcus
f__Enterococcaceae
o__Peptostreptococcales-Tissierellales
f__Peptostreptococcaceae
g__Romboutsia
5 4 3 2 1 0 1 2 3 4 5
LDA SCORE(log10)

5A

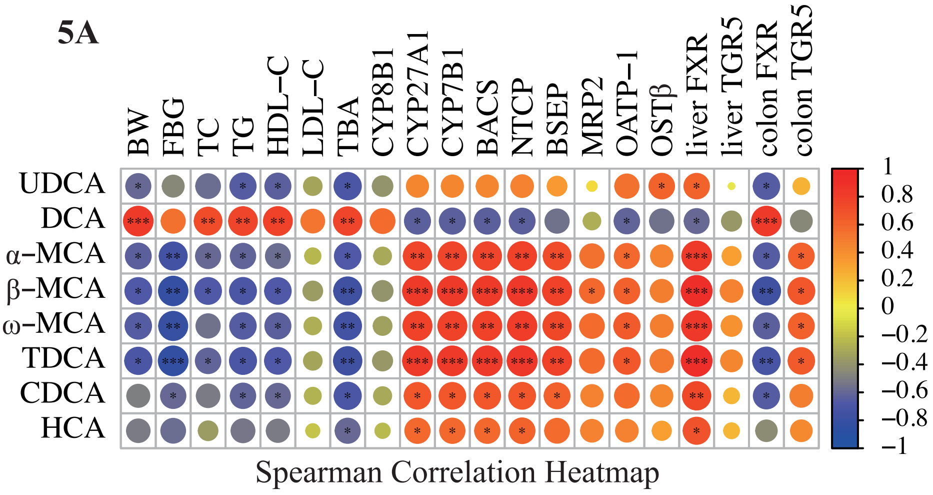

BW
FBG
TC
TG
HDL-C
LDL-C
TBA
CYP8B1
CYP27A1
CYP7B1
BACS
NTCP
BSEP
MRP2
OATP-1
OSTβ
liver FXR
liver TGR5
colon FXR
colon TGR5
UDCA
DCA
α-MCA
β-MCA
ω-MCA
TDCA
CDCA
HCA
1
0.8
0.6
0.4
0.2
0
-0.2
-0.4
-0.6
-0.8
-1
Spearman Correlation Heatmap

5B

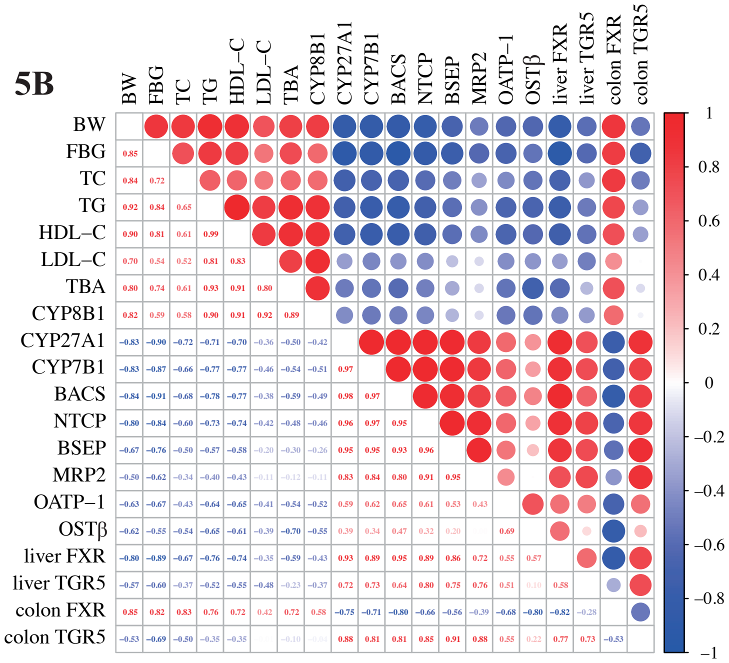

Correlation Corrplot

5C

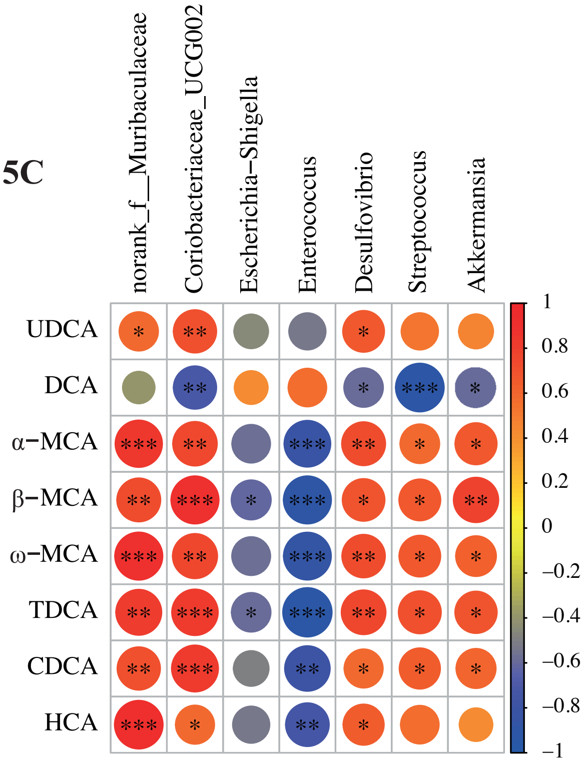

Spearman Correlation Heatmap
